# Editorial: Viruses, genetic exchange, and the tree of life, volume II

**DOI:** 10.3389/fmicb.2023.1271181

**Published:** 2023-08-28

**Authors:** Arshan Nasir, Gustavo Caetano-Anollés, Jean-Michel Claverie

**Affiliations:** ^1^Moderna, Inc., Cambridge, MA, United States; ^2^Evolutionary Bioinformatics Laboratory, Department of Crop Sciences, C.R. Woese Institute for Genomic Biology, University of Illinois at Urbana-Champaign, Urbana, IL, United States; ^3^Structural and Genomic Information Laboratory (UMR7256), Mediterranean Institute of Microbiology (FR3479), IM2B, IOM, Aix Marseille University and CNRS, Marseille, France

**Keywords:** viruses, taxonomy, tree of life, HIV-1, horizontal gene transfer, genetic exchange

The Volume II of “*Viruses, genetic exchange, and the tree of life*” was inspired by the extraordinary success of Volume I of the same topic we published in 2019–20 (Nasir et al., 2019). As of 28 July 2023, articles published in volume I have been viewed >185K times with ~21K downloads indicating a continued interest in the Research Topic. Specifically, Volume I addressed some of the most difficult questions in biology related to viral origins, evolution, and epidemic spread along with how to position microbial/viral taxa onto the broader “Tree of Life”, a metaphor to describe our roots and relatedness. These questions are not purely academic in nature. Only by understanding the past and present of viruses, we can strive to contribute to the development of effective prevention, control, and response strategies, and make tangible contributions to scientific knowledge and public health. Unfortunately, these fundamental questions have remained contentious among scientists for decades, contributing to ongoing debates and differing viewpoints. In Volume II, we continued to receive contributions addressing these outstanding questions. Below we introduce the latest viewpoints dealing with virus outbreaks, taxonomy, origins, and evolution.

Abidi et al. reconstructed the evolutionary history of one of the worse pediatric HIV-1 outbreaks known to mankind. In 2019, hundreds of children tested positive for HIV-1 in the Ratodero area of Larkana district in Pakistan. The unprecedented scale of the HIV-1 outbreak and its spillover to children caused uproar and outrage in the country. The outbreak was initially linked to a private clinic (i.e., a single-source outbreak). Abidi et al. generated *pol* gene sequences from a subset of infected children and analyzed the genetic diversity, transmission clusters, subtype distribution, and drug resistance mutations to trace the origin of the outbreak. The authors identified two common HIV-1 subtypes and four transmission clusters in the outbreak samples providing strong evidence for a multi-source rather than a single-source outbreak. The multiple introductions of the “Larkana strain” was therefore likely a result of poor infection control and prevention strategies that were rampant in the region of Larkana. The article emphasizes the urgent need to implement better hygienic practices across the country, especially in the healthcare clinics, to avoid future outbreaks and to ensure effective control of deadly pathogens.

Caetano-Anollés et al. prompt an urgent reevaluation of the changes recently adopted by the International Committee on Taxonomy of Viruses (ICTV) to (re)-classify viruses. For example, the ICTV abandoned the widely used and intuitive Baltimore system for classification of viruses, introduced the concept of “realms” that are assumed to be monophyletic groups equivalent to “kingdom” level classification in cellular organisms, endorsed virus discovery and naming from metagenomic sequence data alone without the need to validate their existence via culturing, and introduced new names that obscure the scientific history of pathogens, sometimes grouping double-stranded DNA viruses under the higher rank of *Monodnaviria* (hence ssDNA). The authors highlight major problems with these updates such as ignoring the complex evolutionary histories of viruses that are almost impossible to be recovered via traditional sequence or alignment-dependent phylogenetic analyses, the integration of viruses into “holobionts” that invalidate the concept of taxonomic units, assuming monophyly in the absence of a valid test, and confusion about the very definition and nature of viruses, among other challenges. This review is a must read because accurate taxonomic assignment is critical to our understanding and communication about key pathogens and diseases. The burden is now on the ICTV to address the raised criticism.

The research article of Deng et al. explores the use of genomic synteny and codon usage preference as tools for determining genera demarcation within the *Iridoviridae* family of viruses. The researchers analyzed the genomic sequences of various viruses within the family and investigated patterns of conservation of gene order (synteny) and codon usage. Their findings suggest that these genomic features can provide valuable insights into the classification and differentiation of genera. This study holds significant importance for both virology and viral taxonomy. It highlights the potential of genomic analysis techniques in understanding the evolutionary relationships among *Iridoviridae* viruses. Moreover, by utilizing the genomic features of synteny and codon usage preference as novel tools, the study offers a more robust and reliable approach of virus classification and taxonomic delineation. This type of research contributes to a deeper understanding of virus diversity and evolution, thereby enhancing our knowledge of viral biology and paving the way for more effective strategies in virological studies, diagnostics, and antiviral development.

The review article by Harris and Hill opens discussion on important topics that align with the overall theme of the Research Topic. The authors revisit the definition of life and the place of viruses in the overall “Tree of Life”, along with reviewing the popular hypotheses describing the origins of viruses and previous work on investigating the origins of viruses in the light of popular viewpoints. While the article dismisses one of the most promising data-driven investigations of virus origins and evolution by Nasir and Caetano-Anollés (2015) and ignores important follow-up work by the same and other authors revisiting the very definition and nature of viruses (Nasir et al., 2020), it provides a comprehensive examination and synthesis of the evidence supporting the integration of viruses into the tree of life. In particular, the review sheds new light on the role of viruses in shaping biological diversity and evolution, leading to a more holistic understanding of the interconnectedness of all living organisms on Earth.

In his article, Bell revisits the eukaryogenesis hypothesis, initially proposed as early as 2001 (Bell, 2001; Takemura, 2001) then revitalized following the discovery of the first giant viruses (Claverie and Abergel, 2010). The conceptual starting point of this hypothesis is that the strict functional segregation between DNA replication/transcription in the nucleus and protein translation in the cytoplasm perfectly mimics the distribution of roles between the viral factory and the cytoplasm in a cell infected with a large DNA virus. Alternatively, the nucleus can indeed be seen as an intracellular parasite replicating its genome by taking advantage of the metabolic and translational resources confined to the cytoplasm. In the updated and detailed scenario, eukaryotes are now interpreted as superorganisms, where the nucleus descends from a viral factory, the mitochondria from an alpha-proteobacterium, and the cytoplasm and plasma membrane were inherited from an archaeal host whose genome has been lost. This new vision, which restores the central role of viruses in the evolution of life on earth, is sure to provoke further debate.

The Volume II of *Viruses, Genetic Exchange, and the Tree of Life* thus present new and exciting developments in the conceptually and technically challenging field of evolutionary virology. We hope that this Research Topic will become a useful reference for future studies in this research field and will have applications in both basic and applied virology.

## Author contributions

AN: Writing—original draft, Writing—review and editing. GC-A: Writing—original draft, Writing—review and editing. J-MC: Writing—original draft, Writing—review and editing.
